# Limits of Detection of Topically Applied Products in the Skin Using In Vivo Raman Spectroscopy

**DOI:** 10.3390/pharmaceutics16030304

**Published:** 2024-02-22

**Authors:** Cláudio Nico, Tom C. Bakker Schut, Peter J. Caspers, Gerwin J. Puppels

**Affiliations:** 1 RiverD International B.V., 3029 AK Rotterdam, The Netherlands; tbakkerschut@riverd.com (T.C.B.S.); pcaspers@riverd.com (P.J.C.); gpuppels@riverd.com (G.J.P.); 2 Center for Optical Diagnostics and Therapy, Department of Dermatology, Erasmus MC, University Medical Center, 3015 CN Rotterdam, The Netherlands

**Keywords:** stratum corneum, human, non-invasive, quantitative, in vivo, limit of detection

## Abstract

We have developed a method to determine the limit of detection (LoD) for quantitative measurement of exogenous analytes in the outer layer of the human skin by in vivo confocal Raman spectroscopy. The method is in accordance with the guidelines of the International Council for Harmonisation of Technical Requirements for Pharmaceuticals for Human Use that have been adopted by regulatory authorities such as the American Food and Drug Administration and the European Medicines Agency. The method can be applied in silico so that the limit of detection can be assessed before starting a skin penetration study, for example, in areas of pharmaceutical formulation, pharmacokinetics, or toxicokinetics. This can significantly reduce the need for expensive and time-consuming feasibility studies. This paper describes the method to calculate this LoD as well as the experimental and methodological factors that can influence the calculation of the LoD.

## 1. Introduction

Raman spectroscopy is a powerful analytical technique that can be applied to study the molecular composition of the skin and the in vivo permeation of topically applied products in the outermost layer of the skin, the stratum corneum (SC) [1,2,3,4,5,6,7,8,9,10]. This technique is widely used in cosmetic research and in skin permeation studies to quantify the concentrations of analytes in the skin, with particular interest in pharmaceutical formulation, pharmacokinetics, and toxicokinetics.

Despite the large number of papers dedicated to Raman spectroscopic assessment of the skin, few studies have specifically addressed figures of merits of Raman spectroscopy in the quantitative assessment of skin composition and the permeation of analytes in the skin [11].

A key figure of merit for the evaluation of analytical methods is the limit of detection (LoD). The ICH of Technical Requirements for Pharmaceuticals for Human Use (ICH, https://ich.org/ (accessed on 20 February 2024)) has brought together the regulatory authorities and pharmaceutical industry to develop quality guidelines for the validation of analytical procedures, which are now applied by a growing number of regulatory authorities worldwide. The current ICH guideline [12] was developed for univariate techniques such as chromatography. Multivariate analysis methods, such as Raman spectroscopy, are not described in this guideline.

A draft version of the new guideline [13] was published in 2022 and is currently under revision. This new draft Q2(R2) guideline refers to the use of multivariate analysis methods. However, the guideline assumes that it is possible to create a calibration model with samples of known analyte concentrations. Such calibration models can be created for solutes, e.g., for biofluids, where one can prepare samples with different analyte concentrations.

Kichou et al. have used such an in vitro calibration curve [11]. The paper presents a method to determine the analytical performance of Raman spectroscopy for quantification of analytes in the stratum corneum, which is demonstrated by quantitative measurements of the active ingredient resorcinol in isolated stratum corneum sheets. The authors also discuss a range of ex vivo and minimally invasive in vivo techniques to assess the skin penetration of an analyte. Importantly, this paper presents for the first time a LoD of quantitative measurement of an active ingredient in isolated human stratum corneum (ex vivo) by Raman spectroscopy. The authors used a univariate and a multivariate method to determine the LoD. This approach required measurements of stratum corneum samples with a known analyte concentration to establish a calibration curve to convert Raman signals to analyte concentration.

For materials in skin tissue in vivo, which is intrinsically inhomogeneous, the penetration of exogenous analytes cannot be controlled, and thus, creating a calibration model for in vivo skin Raman measurements along these lines is not feasible.

In previous work [1], we have demonstrated how topically applied products can be quantified in vivo by Raman spectroscopy. The method is based on the characteristic that the ratio of the Raman signal intensities of two materials is proportional to the ratio of their mass ratio in a sample. The Raman signal intensity ratio was determined by least-squares fitting. A brief summary of the fitting method is given in the Material and Methods section.

To overcome the problem of a calibration model for in vivo skin samples with accurately known concentrations, we present a method that does not rely on such a calibration model. It uses the method of ‘Limit of Blank’ or ‘Blank determination’ to determine the LoD, which is accepted by the ICH. The limit of the blank is an old and generally applicable method that states that an estimate for the LoD can be obtained by measuring the concentration of an analyte in samples where the analyte is not present and using the resulting analyte distribution to determine a concentration threshold above which it is highly unlikely that the analyte is not present in the sample.

For determining the LoD of a topically applied analyte in skin by Raman spectroscopy, only spectra of untreated skin are needed (blanks). These are then fitted with the set of reference Raman spectra of SC constituents and the spectrum of the analyte (fit model). This can yield non-zero concentrations, even in the absence of the analyte.

Assessment of the factors of influence on the LoD, as determined by the Limit of Blank method, is necessary to enable acceptance of the method in quantitative monitoring of topical delivery by Raman spectroscopy. We have identified five potential factors of influence:

1. The distance to the skin surface,
2. The quality of the fit model,
3. The signal-to-noise ratio of the skin spectrum,
4. The signal strength of the analyte spectrum,
5. Multicollinearity of the analyte spectrum to the fit model.

In this article, we show how the in vivo LoD can be determined using a data set of untreated SC spectra. It illustrates how the five above-mentioned parameters influence the limit of detection. A list of LoD values for materials used in topically applied products is provided, and the results and the limitations of the developed methodology are discussed.

## 2. Materials and Methods

### 2.1. Raman Spectroscopy

The Raman spectra used for this paper were measured on a Raman instrument optimized for in vivo confocal Raman spectroscopy on the human skin (gen2-SCA, RiverD International BV, Rotterdam, The Netherlands) at different locations and operated by different operators. In vivo measurements of the skin were performed with the 785 nm built-in laser and <30 mW laser power on the skin in accordance with the safety guidelines of the International Standard for Laser Devices (IEC 60825-1:2014). The instrument uses a 1.2 numerical aperture oil-immersion objective to focus the laser light on a spot with a diameter <1 µm. Measurements in the skin were conducted with an axial resolution of <5 µm. The spectral resolution is <5 cm^−1^. Signal throughput is >70% from 400 to 4000 cm^−1^, independent of signal polarization. The spectrometer employs a back-illuminated deep-depletion CCD with a quantum efficiency of up to 90% in the detected spectral range.

Raw spectral data were calibrated by the RiverICon 4.1 acquisition software as described in the operator manual (RiverICon, RiverD International BV, Rotterdam, The Netherlands). Briefly, the raw spectral data were corrected for instrument background and offset by subtraction, followed by correction for the wavelength-dependent instrument response by division, and finally, the instrument’s relative wavenumber axis was interpolated on 400–1800 cm^−1^ spectral range.

### 2.2. In Vivo Quantification

To quantify the skin penetration of a topically applied material, Raman spectra are recorded in small, incremental steps at a range of depths in the skin. The spatially resolved concentrations of the topically applied materials are determined from these spectra. For each recorded Raman spectrum, the intensity ratio of the Raman signal contributions of the topically applied material and of the protein fraction of the SC is determined by an ordinary least squares fitting procedure. A set of intensity-normalized reference Raman spectra of SC constituents, together with the spectrum of the topically applied material, are fitted to the Raman spectrum of the skin after treatment with a topical product. The fit coefficients are calculated by the least-squares solution of Ax = b, where A is a matrix of the reference Raman spectra (fit model), x is a vector of fit coefficients, and b is the Raman spectrum of the skin. The ratio of the Raman signal intensities of the material and the protein fraction of the skin at a given depth in the skin is calculated from the fit coefficients. This ratio is proportional to the mass ratio of the material and protein in the skin. The proportionality constant C, which describes the linear relationship between the ratio of the Raman signal intensity and the mass ratio, is determined separately as described in [1].

### 2.3. Skin Spectra

No measurements were performed specifically for the purpose of this paper. Archival data of anonymized Raman profile measurements of untreated skin (blanks) were used for this study. Anonymized data were made available to us by different contract research organizations from a range of studies conducted on adults in the USA and Europe. For each study, informed consent had been obtained in accordance with the local legislation. The data set contained more than 400 Raman profiles from more than 70 subjects measured on the SC of the volar forearm at a depth range of 0–16 µm from the skin surface at 2 μm depth increments. The depth, which is the distance to the skin surface, was determined from the displacement of the focusing actuator in the gen2-SCA relative to the zero position, at which the laser focus was at the skin surface. The optical design of the system is such that a displacement of the objective corresponds to an equal displacement of the laser focus in the skin. Each profile was measured at a different location on the volar forearm, with 5–6 profiles per subject and nine measurements per profile. Each single measurement was performed with an exposure time of 5 s.

The data set was considered representative of the general population, containing skin spectra from subjects of different genders, skin types, and ages.

To investigate the influence of the signal-to-noise ratio on the LoD, we made copies of the data set with simulated exposure times of 4, 3, 2, and 1 s by digitally adding simulated shot noise. The gen2-SCA employs a CCD detector to record shot-noise-limited Raman spectra of the skin. This means that the noise in a spectrum is proportional to the square root of the number of detected photons and, therefore, proportional to the square root of the exposure time. A spectrum with a simulated exposure time of t (1 to 4 s) was calculated from the experimental spectrum with an exposure time of t0 (5 s) by adding noise to the detected photons. The noise was generated as Gaussian white noise with a variance equal to the number of detected photons multiplied by factor (t/t0 − 1).

### 2.4. Analyte Spectra

The quantified analyte spectra used in this article were taken from the component library of SkinTools 3 software (RiverD International B.V., Rotterdam, The Netherlands). This library is a selection of spectra of fragrances, actives, excipients, and solvents that are commonly used in pharmaceuticals and cosmetics and that were obtained from different suppliers. The measurements of each quantified analyte reference spectra were conducted in solution, as described in Caspers et al. [1].

### 2.5. Software

The software to determine the LoD and to investigate the influence of various parameters on the LoD was written in MATLAB (R2017b). For the ordinary least squares analysis, the algorithms from the SkinTools 3 analysis software were used.

### 2.6. Limit of Detection

Here, the Limit of Blank [14,15] method was used to determine the LoD:

LoD = M_b_ + 3∙SD_b_ (1)

where:

LoD = Limit of detection,

M_b_ = mean of (>20) ‘blank’ determinations of the analyte,

SD_b_ = standard deviation of these blank determinations.

This defines the LoD as the concentration level at which the probability is less than 0.5% that the signal is generated by a sample without the analyte present.

Here, a data set of Raman spectra of untreated skin from different subjects, described in the Section 2.3*,* served as “samples without analyte”. For each spectrum of the data set, the ‘blank’ total amount of analyte in the SC concentration for an analyte was calculated by performing a fit of the spectrum with a set of SC model spectra (described below in the section “SC fit model”) and a reference spectrum of the analyte.

This resulted in a distribution of analyte concentrations as a function of depth in the skin. At each depth, the mean concentration, M_b_, and the standard deviation, SD_b_, were determined. The LoD in mg/cm^3^ was calculated as a function of the distance to the skin surface using Equation (1). The mean of the blanks, M_b_, is hereafter referred to as *bias*.

Both the LoD as a function of the skin surface and the LoD for the total amount of analyte in the SC (analyte uptake) are of interest. The LoD for analyte uptake in the SC was calculated by integrating the concentration profiles of the blanks over the thickness of the SC from 0 to 16 µm. The uptake is the mass of analyte per skin surface area (µg/cm^2^). The *uptake LoD* was calculated from the mean and the standard deviation of the distribution of uptakes of the blanks using Equation (1).

### 2.7. SC Fit Model

We refined the previously reported SC fit model [16,17] by adding a spectrum of melanin and replacing the spectra of trans-urocanic acid measured at two different pH values with new trans-urocanic spectra measured at three different pH values.

Melanin may be present in the SC, although in low amounts, and can have a significant contribution to the Raman signal of the skin. A reference spectrum of melanin was derived from in vivo skin spectra from three volunteers without known skin disorders. Spectra measured at the same depth in the skin and containing different levels of melanin were used to create different spectra that only showed melanin features and no features of other skin components. The four difference spectra, with the strongest melanin features and no visible spectral features of other skin constituents, were EMSC-scaled (Extended Multiplicative Signal Correction) to eliminate fluctuating fluorescence backgrounds, using a third-order polynomial as an interfering signal [18]. The EMSC-scaled spectra were averaged to yield a single in vivo melanin spectrum. Following the approach of Huang et al. [19] and Yakimov et al. [20], we fitted the in vivo melanin spectrum with Gaussians to obtain a pure melanin reference spectrum. We used three Gaussians using a Nelder–Mead minimization procedure to model the major sources of variance in the in vivo melanin spectrum, visible in the region 1000–1880 cm^−1^. Smaller signal contributions in the in vivo melanin spectrum in the region 400–1000 cm^−1^ could not with certainty be attributed to melanin and were disregarded.

Trans-urocanic acid (UCA) spectra were obtained at three different pH values, corresponding to the double-protonated, single-protonated, and non-protonated molecules. UCA was purchased from Sigma-Aldrich Chemie (Zwijndrecht, The Netherlands) and dissolved in demineralized water at a concentration of 1 mg/mL. UCA solutions were wrapped in aluminum foil to avoid potential photoisomerization of cis-UCA by UV light. The UCA solution was split into two equal volumes. Both volumes were titrated with pH increments of approximately 0.1 by adding small amounts of a strong base (4 N NaOH) in the pH range of 4.7 to 10 or a strong acid (3 N HCl) in the pH range of 4.7 to 2. In each step, an amount of 10–100 μL of strong acid or base was added. During the experiment, the solution was homogenized with a magnetic stirrer, and pH was continuously monitored with a pH meter until stable. This was typically within 30 to 60 s. A sample of 500 μL was taken from the solution, and a Raman spectrum was recorded with a 20 s exposure time. Contributions from the solvent, water, were subtracted from the calibrated spectra, and spectral outliers due to sampling artifacts or cosmic rays were excluded. A set of independent factors was extracted by Multiple Curve Resolution using three components (MCR-ALS GUI 2.0, Jaumot et al. [21]). The extracted MCR-ALS factors closely resembled the pure spectra at, respectively, pH2, pH5, and pH8 and were labeled accordingly. The factors were normalized by multiplication with their maximum MCR-ALS score, such that the relative contribution of the three MCR-ALS factors to the titration data added up to one at all pH values.

A principal component analysis was performed on large data sets of fit residuals of the SC spectra of the volar forearm. This resulted in the first principal component of the fit residuals (PCFR), which captured over 80% of the variance in the data set of fit residuals. To further reduce the intensity of SC spectrum fit residuals, this PCFR was added to the set of SC fit spectra.

**Figure 1:**
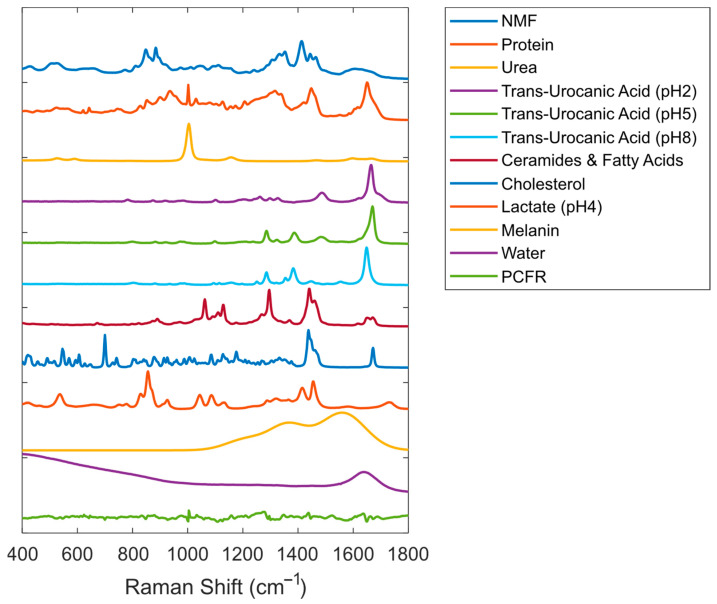
Reference Raman spectra were included in the SC fit model (offset was added to the spectra for visualization purposes). PCFR represents the first principal component of the fit residuals for data from the volar forearm.

A third-order polynomial was included in the fit model to account for the fluorescent background from the skin. The complete SC fit model used in this paper is depicted in Figure 1.

Application of this model to SC spectra resulted in fit residuals of very low intensity (on average, less than 1% of the signal of the skin spectrum being fitted).

### 2.8. Variance Inflation Factor

The effect of adding the analyte spectrum to the SC fit model on the fit model quality was assessed by calculating the variance inflation factor (VIF). The VIF measures the degree of multicollinearity of a spectrum with the other spectra of the fit model, i.e., it evaluates “how different the analyte spectrum is from the skin spectra” [22,23]. The VIF for the analyte spectrum added to the fit model is defined as:

$$VIF=\frac{1}{1-R^{2}}$$

where *R*^2^ is the coefficient of determination of an ordinary least squares linear regression of the analyte spectrum with all other spectra of the fit model. VIF ≤ 5 is widely accepted as an indicator of low multicollinearity [24].

## 3. Results

**Figure 2:**
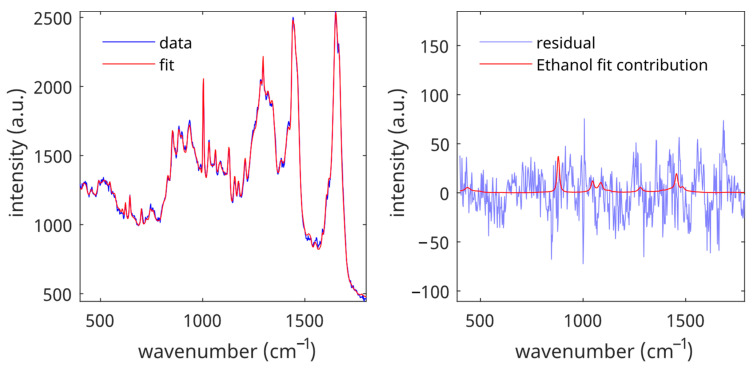
Fit contributions of ethanol in the data set of untreated SC spectra. **Left panel**: spectrum and spectral fit. **Right panel**: fit residual and fit contribution of ethanol for the spectral fit shown in the left panel.

To determine the LoD of an analyte, the reference data set was fitted with the SC fit model spectra and the analyte spectrum. Figure 2 shows a typical example of a blank measurement (spectrum of untreated skin) with ethanol as the analyte. The figure shows that the fit residual is small and that the calculated contribution of ethanol to the blank is in the same order of magnitude as the fit residual.

**Figure 3:**
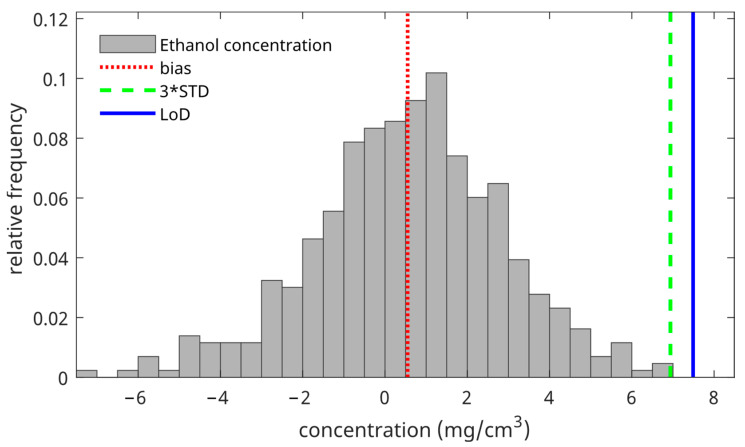
The ethanol concentration distribution of the blank measurements was measured at a depth of 8 µm below the skin surface (gray bars). The red vertical line denotes the bias (mean of the distribution), the green vertical line denotes three times the standard deviation of the distribution, and the blue vertical line denotes the LoD (mean of the distribution + three times the standard deviation of the distribution).

Figure 3 shows the distribution of all calculated concentrations of blank measurements at a depth of 8 µm below the skin surface. From this distribution, we calculated the bias (red line), 3*STD (green line), and the LoD (blue line).

**Figure 4:**
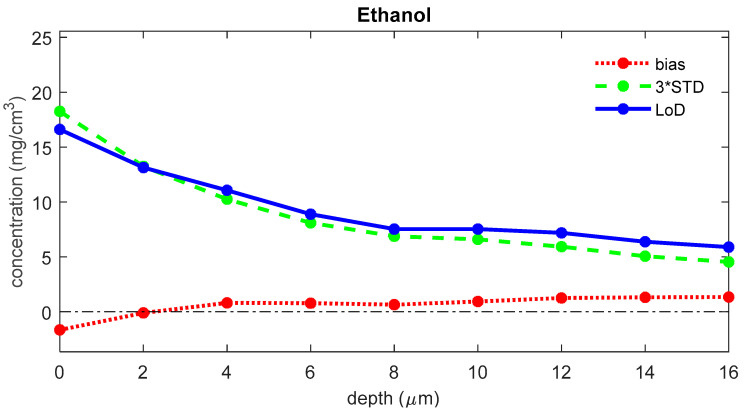
The bias (red), three times the standard deviation (green), and LoD (blue) for the concentration of ethanol as a function of the depth below the skin surface (the least squares fit can yield negative values for concentrations of analytes that are not present in the sample; a negative value represents a concentration below the LoD, meaning that there is no proof of the presence of the analyte).

The LoD was determined for each depth in the skin. Figure 4 shows the LoD, the bias, and three times the standard deviation for ethanol as a function of depth in the skin. For the uptake of ethanol, the bias is 1.1 μg/cm^2^, the 3*STD is 9.5 μg/cm^2^, and the LoD is 10.6 μg/cm^2^.

**Figure 5:**
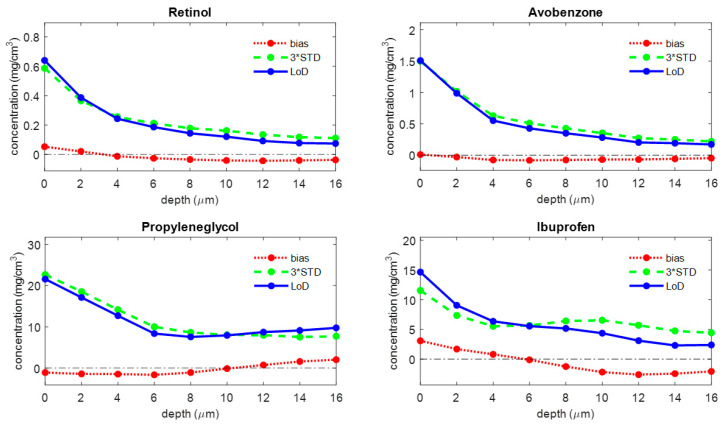
The bias (red), three times the standard deviation (green), and LoD (blue) for four different analytes as a function of depth below the skin surface (the least squares fit can yield negative values for concentrations of analytes that are not present in the sample; a negative value represents a concentration below the LoD, meaning that there is no proof of the presence of the analyte).

Figure 5 shows the LoD curve, the corresponding bias, and 3*STD lines as a function of depth for four different analytes. Figure 4 and Figure 5 demonstrate that the LoD is strongly dependent on the depth of the skin. For all analytes that were assessed in this paper, the bias was generally around zero in the SC. The LoD was generally highest at the skin surface and decreased towards the bottom of the SC. For the uptake of Retinol, the bias is 0.0 μg/cm^2^, the 3*STD is 0.2 μg/cm^2^, and the LoD is 0.2 μg/cm^2^. For the uptake of Avobenzone, the bias is −0.1 μg/cm^2^, the 3*STD is 0.6 μg/cm^2^, and the LoD is 11.9 μg/cm^2^. For the uptake of Propyleneglycol, the bias is −0.5 μg/cm^2^, the 3*STD is 12.4 μg/cm^2^, and the LoD is 11.9 μg/cm^2^. For the uptake of Ibuprofen, the bias is −1.1 μg/cm^2^, the 3*STD is 6.6 μg/cm^2^, and the LoD is 5.5 μg/cm^2^.

Appendix A provides a table with the mean LoD (average of the LoD over the depth range of 0 to 16 μm) and the uptake LoD of all analytes that are included in the quantified component library of SkinTools 3.

**Figure 6:**
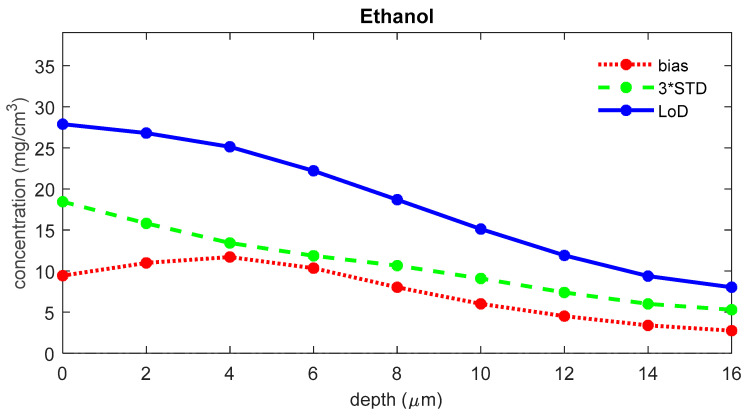
The bias (red), three times the standard deviation (green), and LoD (blue) for ethanol as a function of depth below the skin surface were calculated using a fit model where the NMF spectrum was removed.

To assess the quality of the fit model as a potential factor of influence on the LoD, the LoD was recalculated after the exclusion of the NMF (natural moisturizing factor) spectrum, one of the dominant reference spectra from the SC fit model. Figure 6 shows the LoD of ethanol from the altered fit model, together with the corresponding bias and three times the standard deviation. The bias was significantly increased over the entire depth range in comparison to the results from the complete fit model (see Figure 4). Also, the uptake LoD has a much higher value (26.0 μg/cm^2^), while the bias is 12.1 μg/cm^2^, and the 3*STD is 13.9 μg/cm^2^.

**Figure 7:**
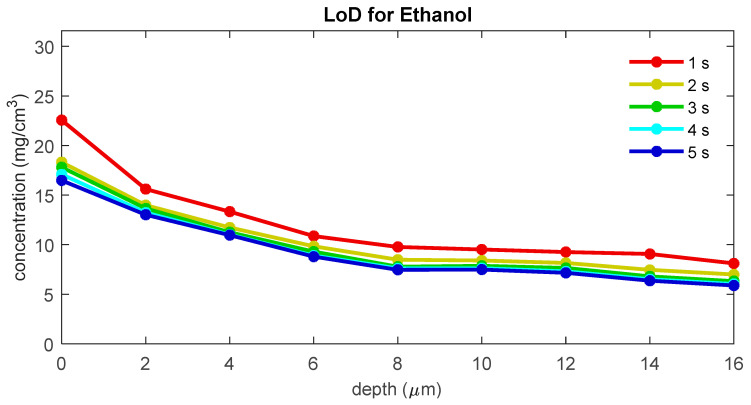
The LoD for ethanol as a function of depth below the skin surface for different exposure times.

To evaluate the influence of the signal-to-noise ratio on the LoD, we recalculated the LoD for ethanol using copies of the data set with different simulated exposure times. Figure 7 shows the LoD for blank spectra with simulated exposure times of 1 to 5 s. The figure shows that LoD and uptake LoD increase with decreasing exposure time. For the uptake of ethanol, the LoD is 12.4 μg/cm^2^ for 1 s measurements, 10.9 μg/cm^2^ for 2 s measurements, 10.5 μg/cm^2^ for 3 s measurements, 10.4 μg/cm^2^ for 4 s measurements, and 10.5 μg/cm^2^ for 5 s measurements.

**Figure 8:**
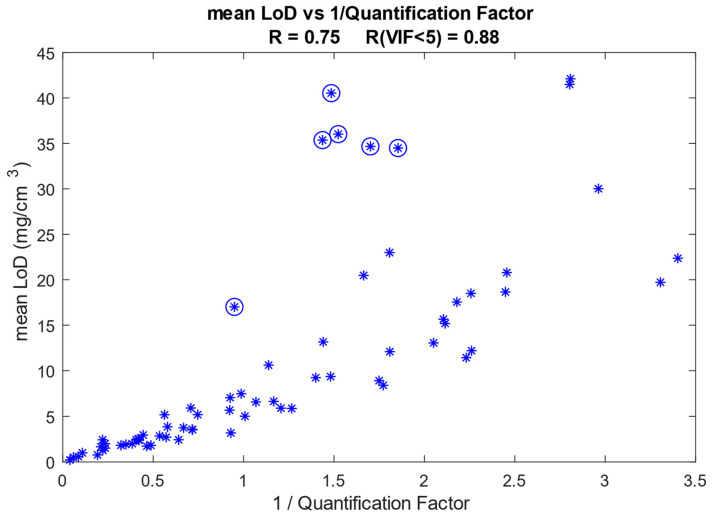
Mean LoD as a function of the inverse of the quantification factor (QF). Each asterisk represents an analyte. The asterisks enclosed in circles represent analyte spectra that have a VIF higher than 5 with our SC fit model. The Pearson correlation coefficient is 0.75 if all analytes are included and increases to 0.88 when the analytes that have a VIF of five or higher are excluded.

We have assessed the influence of the Raman signal strength of the analyte on the calculated LoD. The Raman signal strength is reflected by the quantification factor (QF), which is the proportionality constant between the Raman signal intensity ratio and the mass ratio of the analyte and protein. Figure 8 shows the relation between the mean LoD (averaged over the depth range between 0 and 16 µm) and the inverse of the QF for all quantified analytes currently available in our SkinTools library. The LoD has a correlation coefficient (R) of 0.75 with the inverse of QF.

The shape of the Raman spectrum of the analyte determines its collinearity with the reference spectra of the SC constituents in the fit model. The degree of collinearity of the analyte spectrum with the SC fit model is given by the VIF, as explained in the Materials and Methods section. In Figure 8, the points that correspond to analyte spectra with a VIF > 5 (some collinearity with the SC fit model) are indicated with circles. After excluding these points, the correlation coefficient increases to 0.88.

**Figure 9:**
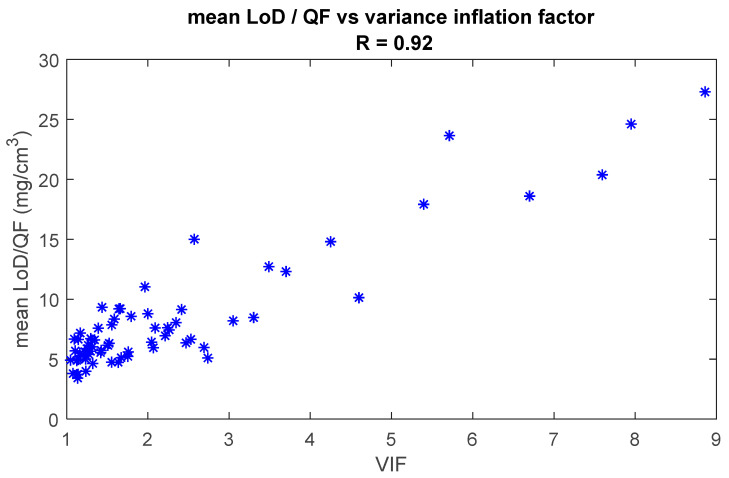
Mean LoD is divided by the quantification factor as a function of the variance inflation factor (VIF). Each asterisk represents a quantified analyte. The Pearson correlation coefficient is 0.92.

Figure 9 shows the mean LoD/QF as a function of the VIF for all analytes referenced in this paper. The mean LoD was divided by the quantification factor to compensate for the differences in Raman signal strength. The figure shows that there is a strong correlation (R = 0.92) between the LoD/QF and the VIF. This clearly illustrates that the lack of orthogonality between the spectrum of an analyte and the spectra of the skin fit model will significantly increase the LoD of the analyte.

## 4. Discussion

For in vivo detection of topically applied products in the skin using Raman spectroscopy, the LoD is an important parameter that will determine the feasibility of a skin penetration study for a given concentration of the applied analyte and a given set of measurement parameters. In this article, we describe a novel method to determine the LoD, based on the ‘Limit of Blank,’ for detecting exogenous analytes in the human SC using in vivo confocal Raman spectroscopy. A practical advantage of this method is that it can be applied in silico using a data set of spectra from untreated skin. By doing so, the LoD of an analyte can be assessed independently before the start of an in vivo skin penetration study. This can significantly reduce the need for expensive and time-consuming feasibility studies.

Our least squares fitting method, described in [1], translates a multivariate spectrum into fit coefficients for the different components of the fit model. If the component is quantified, the fit coefficient can be translated into a concentration. This concentration can be regarded as a univariate result for which the standard ICH guidelines [12] to determine the LoD can be used. The fact that we cannot use a multivariate calibration approach, as proposed by the new draft guidelines [13], is caused by the lack of a gold standard, i.e., reliable reference concentrations for our high spatial resolution in vivo skin measurements.

The lack of a gold standard also prevented the use of another much-used definition of the LoD that is defined in the EP17 guideline, Protocols for Determination of Limits of Detection and Limits of Quantitation, of the Clinical and Laboratory Standards Institute [14], and described in detail by Armbruster and Pry [25]. This definition states that the LoD should be defined as the minimum concentration that can be detected reliably with a sufficient degree of confidence, thereby also including the variance of low-concentration samples:

LoD = LoB +1.645*SD_low concentration samples_

where LoB = mean_blanks_ + 1.645*SD_blanks_

Although this is a stricter definition, we also cannot use this definition because we cannot obtain a gold standard estimate for low-concentration samples from in vivo skin measurements. However, we expect that the two definitions will not give very different LoD values because both the standard deviation of the low-concentration samples and the standard deviation of the blanks will be mainly determined by the variance in skin composition.

Given this limitation, the Limit of Blank approach described in the article can provide a good estimate for the lower detection limit. The results obtained with our least squares fit method and our SC fit model show that this limit is in the range of 0.1 to 100 mg/cm^3^ for the concentration and in the range of 0.1 to 100 µg/cm^2^ for the uptake for all quantified analytes in our library.

The LoD consists of two terms: a bias and a variance term. The bias is generally around zero over the whole depth range, and the variance is generally highest at the skin surface and somewhat lower for the deeper layers of the SC (Figure 4 and Figure 5). This higher variation near the surface is mostly due to higher subject-to-subject variance in the surface layer of the skin.

The signal-to-noise ratio of the measured skin spectrum can be a limiting factor for achieving a low LoD. A lower signal-to-noise ratio can lead to a larger variance in the blank concentrations and, therefore, a higher LoD. For spectra measured with a gen2-SCA, the LoD appears to be only limited by the exposure time and, therefore, the signal-to-noise of the spectra for low exposure times. From the results shown in Figure 7, one can conclude that only spectra with exposure times of less than 3 s show a significant increase in the height of the LoD. A standard exposure time of 5 s provides a safe margin to prevent signal-to-noise from becoming a limiting factor for the LoD.

The SC fit model is a crucial part of our methodology. The fit model should describe all spectral variance that can be encountered in the SC so that the fit results have low residuals. As the molecular composition of the SC changes with depth, the quality of the fit model can also vary with depth in the skin, and consequently, the LoD can be dependent on the measurement depth. If the fit residuals of the fitted spectra are significant in size, any analyte spectrum that is added to the fit model will be used to cover as large a portion of this residual as possible. If the fit residuals show a systematic and structured variance because one or more components are missing from the fit model, this can lead to a significantly higher bias, as demonstrated in Figure 6, where we left out the NMF spectrum of our SC fit model. On the other hand, the number of spectra in the fit model should be kept to a minimum, and the fit spectra should be as non-collinear as possible to prevent any analyte spectrum that is added to the fit model from being largely fitted with the spectra of the fit model. We optimized our SC fit model by adding the first principal component of a large data set of SC fit residuals to the fit model and by keeping the VIF factors of the spectra in the fit model as low as possible.

### Limitations and Perspectives

We did not investigate the dependence of the LoD on the extent of the wavenumber range used for analysis. We only investigated the factors that influence the height of the LoD for the wavenumber range of 400–1800 cm^−1^. However, the same methodology can be used for techniques such as CARS (coherent anti-Stokes Raman spectroscopy), which only uses a limited part of the Raman spectrum to identify the spectral region bands that would give the lowest possible LoD.

In the results described above, the LoD was determined using a large, anonymized data set of spectra of untreated skin from multiple studies, which encompass both genders, a diversity of skin types, and a wide age range. The diversity included in this database grants sufficient variance to calculate a general LoD. In a penetration study, this LoD can be used for all test subjects and would eliminate the necessity of doing baseline measurements, which is now common practice. In principle, one can achieve even lower LoD values for a single test subject by doing baseline measurements for each subject. Because the variance in skin composition within a single subject is smaller than the variance encountered in a large data set with many subjects, one can achieve lower LoD values at a personal level at the expense of having to do baseline measurements.

Although the current approach was developed for exogenous analytes, the method can, in principle, also be used for endogenous skin components, which may be ingredients of a product (e.g., lactate, urea, ceramide). The fact that these analytes are naturally present in the skin will lead to large bias and variance terms in the LoD and, therefore, to a high LoD. However, if concentrations of the analyte are encountered that are above this LoD, they must be due to penetration of the product.

## 5. Conclusions

We demonstrated how various parameters determine the height of the LoD. Some parameters, like the shape and Raman strength signal of the analyte spectrum, cannot be optimized but have a strong influence on the LoD. Using our library of quantified components, we showed that the height of the LoD has a high correlation with the inverse of Raman signal strength (Figure 8). In other words, the stronger the Raman signal of the analyte, the lower the LoD will be. We also showed that the LoD shows a strong correlation with the degree of collinearity of the analyte spectrum with the fit model (Figure 9), meaning that the more equal the analyte spectrum is to the spectra in the fit model, the higher the LoD will be.

By introducing this innovative in silico method, this paper aims to contribute to the advancement of non-invasive analytical techniques in the field of dermatological research. Understanding the impact of experimental parameters on LoD will enable researchers to design more robust and effective experiments while optimizing resources and reducing their reliance on extensive in vivo testing.

## Data Availability

Data are contained within the article.

## Appendix A

**Table A1:** Mean LoD (mg/cm^3^) and uptake LoD (µg/cm^2^) for various analytes.

	Analyte	Mean LoD	Uptake LoD
1	1-Propanol	13	14.8
2	1-Tetradecanol	35.4	36.3
3	2-Butanone	12.1	15.4
4	2-PhenoxyEthanol	3.7	4.2
5	3-Methyl salicylic acid	5.9	6.3
6	4-Methyl salicylic acid	5.2	6.1
7	Acetone	6.6	7.8
8	Adenosine	7.5	7.4
9	Avobenzone	0.5	0.5
10	Benzaldehyde	1.3	1.5
11	Benzoin	2	2.2
12	Benzyl Alcohol	2.4	2.9
13	Benzylparaben	2.5	2.8
14	Bisfenol	2.4	2.6
15	Butylene glycol	18.7	20.6
16	Butylene Glycol Dicaprylate	34.7	35.8
17	Caffeine	3.6	4
18	Citric Acid	22.4	26.6
19	Coumarin	0.8	0.8
20	Cyclohexane	1.8	1.9
21	Diclofenac	5.7	5.9
22	Dimethyl Sulfoxide	1.7	1.8
23	Dipropylene Glycol	20.8	24.5
24	Ethanol	9.4	10.6
25	Ethylhexyl triazone	0.6	0.6
26	Ethylparaben	1.8	1.8
27	Eugenol	6.6	7.4
28	Geraniol	17	20
29	Glucose	19.7	20.1
30	Glycerol	42.1	51.4
31	Glycolic acid	12.2	13.6
32	Hydrocortisone	7	6.9
33	Hydroquinone	2.7	2.9
34	Ibuprofen	5.9	5.5
35	Isobutylparaben	2.4	2.4
36	Isopropanol	3.2	2.9
37	L-Alanine	8.9	8.6
38	L-Arginine	41.5	51.3
39	L-Ascorbic Acid	10.6	12.6
40	Laurocapram	20.5	21.1
41	L-Citrulline	30	33.9
42	Levulinic acid	15.2	18.4
43	Lidocaine	9.2	10.4
44	Menthol	5.9	6.4
45	Methanol	8.4	8.8
46	Methyl salicylate	3	3.8
47	Methylparaben	1.9	2
48	Myritol Oil	34.5	35.7
49	Nicotinamide	3.5	4
50	Nicotine	5	5.7
51	Octocrylene	1.5	1.7
52	Octyldodecanol	40.5	40.6
53	Oleic Acid	36	36.1
54	Oxybenzone	2	2.2
55	Phenethyl Alcohol	3.9	4.4
56	Polyethylene glycol 300	15.7	18.8
57	Propanediol	17.5	20.9
58	Propyleneglycol	11.4	11.9
59	Propylparaben	2.5	2.5
60	Retinol	0.2	0.2
61	Salicylic acid	2.9	3.2
62	Sodium Benzoate	3.1	3.7
63	Sodium Dodecyl Sulfate	23	25.6
64	Testosterone	5.2	4.9
65	trans-Cinnamic Acid	1	1.2
66	Tripropylene glycol	18.5	21.6
67	Uvinul A Plus	2.4	2.4
68	Vanillin	1.7	1.8
69	α-Tocopherol	13.2	13.5
